# Research on a Predictive Model for Microalbuminuria in Type 2 Diabetes Based on Machine Learning and SHAP Analysis

**DOI:** 10.1155/ije/6356560

**Published:** 2026-03-01

**Authors:** Zixuan Liu, Zhuolin Zhou, Yu Sun, Xiaotian Du, Haixia Zhang, Cheng Ji

**Affiliations:** ^1^ Department of Pharmacy, Nanjing Drum Tower Hospital, Affiliated Hospital of Medical School, Nanjing University, Nanjing, 210008, China, nju.edu.cn; ^2^ Department of Drug Clinical Trials, Affiliated Wuxi People’s Hospital of Nanjing Medical University, Wuxi, 214023, China, njmu.edu.cn; ^3^ Department of Pharmacy, China Pharmaceutical University, Nanjing Drum Tower Hospital, Nanjing, 211198, China, nju.edu.cn; ^4^ Department of Pharmacy, Nanjing Drum Tower Hospital Clinical College of Nanjing University of Chinese Medicine, Nanjing, 210023, China, nju.edu.cn; ^5^ University of International Business and Economics, Beijing, 100029, China, uibe.edu.cn

**Keywords:** albuminuria, algorithms, electronic medical records, machine learning, type 2 diabetes mellitus

## Abstract

**Background:**

Type 2 diabetes mellitus (T2DM) is associated with kidney damage, with microalbuminuria (MAU) serving as an early marker indicating the risk of progression to severe renal and cardiovascular complications, and there is an urgent need for effective prediction tools to identify MAU risk in T2DM patients and prevent adverse outcomes. This study aims to develop a machine learning–based model to enhance the early identification of high‐risk individuals and facilitate timely, personalized interventions.

**Methods:**

The electronic medical records of 4170 patients were retrospectively extracted from the diabetes special database of Nanjing Drum Tower Hospital (Ethics approval number: 2021‐403‐02). The data were divided into training and testing sets (8:2 ratio), and random forest–based recursive feature elimination method was employed to identify the most pertinent input variables for the predictive model. Five machine learning models were applied to predict the progression to MAU. The Shapley additive explanations (SHAP) values were applied for model interpretation to assess feature contributions. Ten features were selected for the construction of a prediction model.

**Results:**

For predicting the progression to MAU, the Light GBM model demonstrated the best performance (AUC 0.85, 95% CI 0.82–0.88). By analyzing the Shapley values of the model outputs, we identified the key risk factors for predicting the diagnosis of MAU at both the cohort and individual levels.

**Conclusions:**

This study developed an interpretable machine learning model to predict MAU in T2DM patients, enabling effective risk stratification and identification of high‐risk individuals based on baseline data to guide personalized clinical interventions and optimization of treatment.

## 1. Introduction

Type 2 diabetes mellitus (T2DM) is a chronic metabolic disorder characterized by insulin resistance and inadequate secretion, leading to persistent hyperglycemia, which may result in kidney damage, such as glomerulosclerosis and increased urinary albumin [1]. Microalbuminuria (MAU), defined as a urinary albumin excretion of 30–300 mg over 24 h or a urinary albumin‐to‐creatinine ratio (UACR) of 30–300 mg/g, serves as an early marker of kidney damage, signaling the potential progression of T2DM into severe renal diseases and cardiovascular disorders. MAU is a critical indicator of the potential evolution from T2DM to clinically significant proteinuria and renal impairment in patients [2]. Studies indicate that some patients have developed MAU or even macroalbuminuria (CAU) at the initial diagnosis of T2DM. In the absence of specific interventions, 20%–40% of patients with MAU may gradually progress to CAU, and about 20% of those with CAU will develop end‐stage renal disease within 20 years [3], accompanying high morbidity and mortality rates, with significantly increased treatment costs [4]. Additionally, MAU is also considered to increase the risk of cardiovascular diseases, hypertension, and other complications. Consequently, MAU is regarded as a critical prognostic marker for the onset of diabetic nephropathy and the subsequent development of cardiovascular diseases in patients with T2DM [5]. Identifying the risk factors associated with MAU in T2DM patients and implementing targeted interventions are essential for delaying, or even potentially reversing early diabetic kidney damage and thereby preventing end‐stage renal disease.

Machine learning (ML) methodologies confer significant benefits over conventional statistical models due to “their flexibility,” proficiency in handling complex nonlinear relationships, and capacity for processing large‐scale data. This has been driving research advancements across various clinical domains, particularly in clinical radiology [6], pharmacokinetics [7], and oncology [8]. However, there remains a paucity of information regarding predictive models for the prognosis and complications [9, 10] of lifestyle‐related diseases such as T2DM [11]. Few predictive tools that can be integrated into clinical practice are available to accurately stratify the risk of MAU in diagnosed T2DM patients. Accordingly, this study aims to develop a risk prediction model using ML techniques to facilitate the early and accurate identification of high‐risk MAU individuals among T2DM patients, enabling timely personalized intervention strategies.

## 2. Methods

### 2.1. Data Source and Patient Selection

The data for this study were retrospectively collected and organized from the specialized diabetes database on the YiduCloud platform, which is affiliated with the Nanjing Drum Tower Hospital in Jiangsu. The clinical data were gathered from patients who visited the Department of Endocrinology between August 2010 and June 2023.

The study included patients from the specialized diabetes database who met the following criteria: (1) baseline diagnosis of T2DM with normal urinary protein excretion; (2) at least two diagnoses of diabetic MAU made at least 6 months after the baseline; and (3) patients who had never been diagnosed with diabetic MAU. The diagnosis of T2DM was based on the 1999 World Health Organization criteria [12]: fasting blood glucose ≥ 7.0 mmol/L or 2‐h postprandial blood glucose ≥ 11.1 mmol/L.

Proteinuria was categorized into three stages based on the UACR: UACR < 30 mg/g was considered normal albuminuria, 30 mg/g < UACR < 300 mg/g was defined as MAU, and UACR > 300 mg/g was considered CAU [13].

The exclusion criteria were as follows: (1) age < 18 years; (2) history of malignant tumors; (3) severe liver dysfunction; and (4) the presence of other conditions that could affect diabetic MAU, such as acute glomerulonephritis or acute kidney injury.

### 2.2. Handling of Missing and Outlier Data

To manage missing data, the R packages “dplyr” and “tidyr” were utilized to assess the proportion of missing values across the dataset. Variables with more than 20% missing data were excluded from the analysis. For variables with a missing proportion of ≤ 20%, the “mice” package was employed to perform multiple imputation.

In dealing with outliers, outliers were then identified using box plots and the interquartile range (IQR) method. Given that certain biochemical markers may exhibit abnormal values due to the disease state, the study took a conservative approach to ensure data integrity. Instead of outright rejecting all outliers, a manual review process was conducted, considering the patients’ actual clinical conditions before excluding any outliers from the analysis.

### 2.3. Variable Selection

In this study, 32 candidate predictive variables were identified based on clinical expertise and a review of published literature to develop the MAU risk prediction model. The continuous variables included age, duration of diabetes, white blood cell count, red blood cell count, neutrophil‐to‐lymphocyte ratio (NLR), hemoglobin (Hb), platelet count (PLT), serum albumin (ALB), serum creatinine (Scr), uric acid (UA), total cholesterol (TC), triglyceride (TG), high‐density lipoprotein cholesterol (HDL‐C), low‐density lipoprotein cholesterol (LDL‐C), fasting plasma glucose (FPG), fasting C‐peptide, glycated hemoglobin (HbA1c), estimated glomerular filtration rate (eGFR), serum potassium, sodium, calcium, phosphorus, and alkaline phosphatase (ALP). The binary variables and their coding rules included gender (male coded as 1, female coded as 0), presence of hypertension, and use of specific medications such as metformin, insulin, glucagon‐like peptide‐1 receptor agonists, sodium‐glucose cotransporter‐2 inhibitors (SGLT‐2is), angiotensin‐converting enzyme inhibitors, angiotensin II receptor blockers, and statins (use coded as 1, nonuse coded as 0). For feature selection, a recursive feature elimination method based on random forests was employed to filter the input variables for the predictive model.

### 2.4. Outcome Definition

The binary outcomes of the predictive model are defined as whether T2DM patients progress to MAU, with progression encoded as 1 and nonprogression as 0.

### 2.5. Model Construction

Modeling and statistical analysis were conducted using R software Version 4.3.0. Data were randomly split into training and testing sets at a ratio of 8:2. The training set was utilized for training ML algorithms and fitting models. The trained models were then applied to the testing set, and performance metrics were calculated based on the discrepancies between predicted and actual outcomes to assess the predictive performance and classification efficacy of the models. This study employed five ML models: logistic regression (LR), decision tree (DT), support vector machine (SVM), light gradient boosting machine (Light GBM), and extreme gradient boosting (XGBoost).

For continuous variables that followed a normal distribution and had homogenous variances, descriptions were given using mean ± standard deviation (*x* ± *s*), and the *t*‐test was used for comparisons between two groups. For continuous variables that do not follow a normal distribution or have heterogeneous variances, descriptions are given using the median and IQR, and the Mann–Whitney U nonparametric test is used for comparisons between two groups. Categorical data are described using frequencies or percentages, and comparisons between groups are conducted using the chi‐square test. In all statistical tests, a *p* value less than 0.05 is considered statistically significant, indicating a meaningful difference.

### 2.6. Model Evaluation

The performance of the models is assessed by generating a confusion matrix and using metrics such as the area under the receiver operating characteristic curve (AUC), accuracy, recall, specificity, positive predictive value (PPV), negative predictive value (NPV), and F1 score [14]. A higher AUC value [15] indicates better discriminative ability of the predictive model. The model with the best performance is selected for final interpretation.

### 2.7. Model Visualization and Interpretation

We employ the SHAP (Shapley additive explanations) framework to assign an importance value to each feature for a specific prediction [16]. This method quantifies the contribution of each feature in the model to the final prediction outcome and utilizes a predictive model built on all possible combinations of feature subsets, including the given feature.

## 3. Results

### 3.1. Patient Characteristics

The original dataset for this study consisted of 5322 patients. After addressing missing and outlier values, the study included 4170 patients with a median follow‐up duration of 2 years; details are provided in Table 1. During the follow‐up period, 1063 patients (25.5%) progressed to MAU, while 3107 patients (74.5%) did not progress. The median age of the patients was 58 years (IQR 48–68), with the age of patients who progressed to MAU being significantly higher at 63 years (IQR 52–75) compared to those who did not progress (*p* < 0.001).

**TABLE 1:** Baseline information table for Type 2 diabetes patients.

Variables	Total (*n* = 4170)	Non‐MAU (*n* = 3107)	MAU (*n* = 1063)	*p* value
Sex, *n* (%)				< 0.001
Female	1649 (40)	1168 (38)	481 (45)	
Male	2521 (60)	1939 (62)	582 (55)	
Age, years	58 (48, 68)	57 (48, 66)	63 (52, 75)	< 0.001
Duration of diabetes, years	10.5 (1.20)	8.5 (3.14)	12 (6.18)	< 0.001
WBC, 10^9^/L	6.12 (5.2, 7.3)	6.1 (5.2, 7.3)	6.4 (5.3, 7.4)	< 0.001
RBC, 10^9^/L	4.56 (4.22, 4.96)	4.57 (4.29, 4.95)	4.48 (3.96, 5)	< 0.001
NLR	1.93 (1.55, 2.73)	1.83 (1.53, 2.56)	2.22 (1.65, 3.07)	< 0.001
Hb, g/L	137 (126, 148)	139.13 (129, 149)	130 (121, 142)	< 0.001
PLT, 10^9^/L	193.6 (162, 237)	194.25 (164, 241)	186 (155, 217.5)	< 0.001
ALB	43.1 (40.4, 45)	43.1 (40.7, 44.9)	43 (39.7, 45.2)	0.042
Ca, mmol/L	2.42 (2.32, 2.51)	2.42 (2.33, 2.51)	2.41 (2.31, 2.51)	0.136
Mg, mmol/L	4.1 (3.86, 4.35)	4.1 (3.86, 4.36)	4.12 (3.84, 4.34)	0.922
Na, mmol/L	139.9 (138, 141.5)	140 (138.1, 141.5)	139.6 (137.75, 141.5)	0.081
P, mmol/L	1.11 (1.01, 1.23)	1.12 (1.02, 1.23)	1.1 (1, 1.22)	0.017
Scr, umol/L	57.15 (45, 71)	58 (46, 71)	57 (43, 73.5)	0.534
UA, umol/L	323.5 (267, 388)	323 (267, 387)	325 (265, 391)	0.783
TC, mmol/L	4.38 (3.66, 5.16)	4.39 (3.7, 5.17)	4.36 (3.59, 5.14)	0.108
TG, mmol/L	1.3 (0.88, 1.97)	1.28 (0.88, 1.96)	1.37 (0.89, 2.02)	0.047
HDL‐C, mmol/L	1.12 (0.91, 1.38)	1.1 (0.91, 1.38)	1.12 (0.91, 1.4)	0.566
LDL‐C, mmol/L	2.44 (1.85, 3.07)	2.46 (1.89, 3.1)	2.34 (1.74, 2.96)	< 0.001
FPG, mmol/L	7.29 (6.01, 9.35)	7.19 (5.96, 9.26)	7.5 (6.2, 9.54)	0.006
ALP, U/L	70.5 (58.1, 86.9)	70.5 (58.2, 86.75)	70.5 (57.5, 87.3)	0.728
Fasting C‐peptide, pmol/L	725.8 (530, 952.9)	719.5 (522.2, 938.5)	750.8 (555.45, 1011)	< 0.001
HbA1c, %	7.2 (6.3, 8.7)	7.3 (6.3, 8.7)	7.1 (6.3, 8.4)	0.033
eGFR, mL/min/1.73 m^2^	114.3 (95.9, 137.1)	115.4 (98.1, 137.9)	112.6 (86.1, 135.3)	< 0.001
HTN, *n* (%)				0.33
No	3367 (81)	2520 (81)	847 (80)	
Yes	803 (19)	587 (19)	216 (20)	
Metformin, *n* (%)				0.473
No	3074 (74)	2281 (73)	793 (75)	
Yes	1096 (26)	826 (27)	270 (25)	
Insulin, *n* (%)				< 0.001
No	2598 (62)	1747 (56)	851 (80)	
Yes	1572 (38)	1360 (44)	212 (20)	
SGLT‐2i, *n* (%)				0.512
No	3960 (95)	2946 (95)	1014 (95)	
Yes	210 (5)	161 (5)	49 (5)	
GLP‐1R, *n* (%)				0.564
No	4109 (99)	3064 (99)	1045 (98)	
Yes	61 (1)	43 (1)	18 (2)	
ARB, *n* (%)				< 0.001
No	3765 (90)	2840 (91)	925 (87)	
Yes	405 (10)	267 (9)	138 (13)	
ACEi, *n* (%)				0.049
No	4040 (97)	3000 (97)	1040 (98)	
Yes	130 (3)	107 (3)	23 (2)	
Statin, *n* (%)				0.012
No	3340 (80)	2460 (79)	880 (83)	
Yes	830 (20)	647 (21)	183 (17)	

*Note:* ALB, albumin; Ca, serum calcium; Mg, serum magnesium; Na, serum sodium; P, serum phosphorus; Scr, serum creatinine; HTN, hypertension; Hb, hemoglobin; TG, triglyceride.

Abbreviations: ACEi, angiotensin‐converting enzyme inhibitors; ALP, alkaline phosphatase; ARB, angiotensin II receptor blockers; eGFR, estimate glomerular filtration rate; FPG, fasting plasma glucose; GLP‐1RA, glucagon‐like peptide‐1 receptor agonists; HbA1c, hemoglobin A1C; HDL‐C, high‐density lipoprotein cholesterol; LDL‐C, low‐density lipoprotein cholesterol; NLR, neutrophil‐to‐lymphocyte ratio; PLT, platelet count; RBC, red blood cell count; SGLT‐2is, sodium‐glucose cotransporter‐2 inhibitors; TC, total cholesterol; UA, urine acid; WBC, white blood cell count.

### 3.2. Feature Selection Results

**FIGURE 1:**
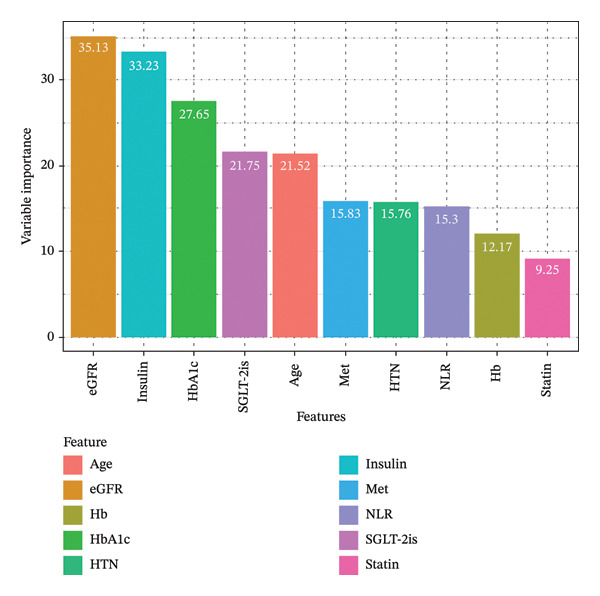
Feature importance ranking. Abbreviations: eGFR, estimated glomerular filtration rate; Hb, hemoglobin; HbA1c, hemoglobin A1C; HTN, hypertension; Met, metformin; NLR, neutrophil‐to‐lymphocyte ratio; SGLT‐2is, sodium‐glucose cotransporter‐2 inhibitors.

Recursive feature elimination using a random forest algorithm with fivefold cross‐validation was utilized to determine the variables to be included. Based on the importance ranking of features, the final selection included the following 10 variables: age, Hb, NLR, HbA1c, eGFR, presence of hypertension, insulin usage, metformin, SGLT‐2is, and statins, as the most relevant features for input into the model (Figure 1). Among these, the baseline glomerular filtration rate in patients with T2DM showed the strongest correlation with progression to MAU.

### 3.3. Model Development, Validation, and Evaluation

The 10 variables most closely associated with MAU risk in T2DM patients were used as input variables to develop predictive models using five different ML algorithms. A total of 3240 patients were used as the training set for model development, while 809 patients comprised the testing set to validate model performance, with the MAU status of these test patients being known. Given the substantial data volume and the lack of externally diverse data from those used in modeling, this study employed a random split for internal validation.

**FIGURE 2:**
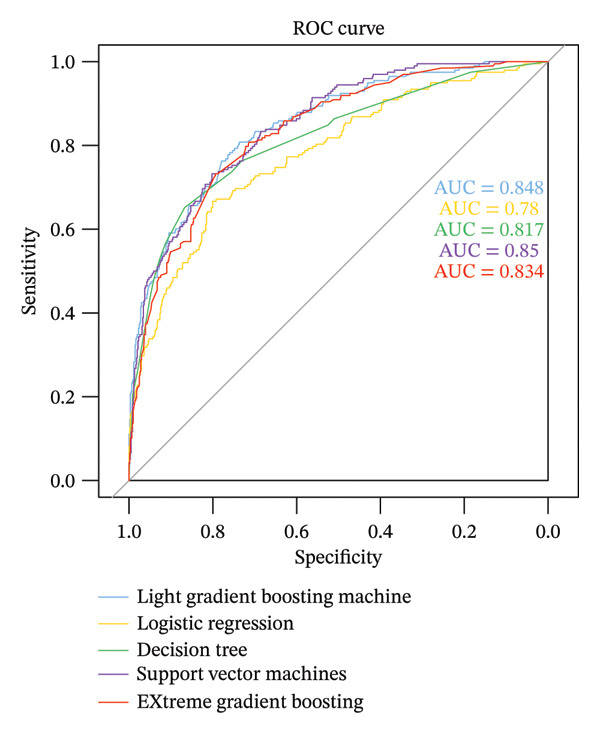
ROC curve plot for machine learning algorithm prediction model. Abbreviations: ROC, receiver operating characteristic curve; AUC, area under the curve.

The performance evaluation results of the models generated by the five ML algorithms are presented in Table 2. The accuracy, specificity, and recall were high across all five models, indicating their effective predictive capability for the risk of developing MAU. The receiver operating characteristic (ROC) curves for each model are shown in Figure 2, with the Light GBM model exhibiting the highest AUC value (0.85, 95% CI 0.82–0.88), indicating it had the best performance.

**TABLE 2:** Performance evaluation table for five machine learning algorithms.

Model	AUC (95% CI)	Cutoff	Accuracy	Recall	Specificity	PPV	NPV	F1 score
LR	0.78 (0.74–0.82)	0.304	0.74	0.67	0.76	0.48	0.88	0.56
DT	0.82 (0.79–0.86)	0.275	0.82	0.65	0.87	0.61	0.88	0.63
SVM	0.85 (0.82–0.88)	0.161	0.76	0.74	0.78	0.51	0.90	0.60
Light GBM	0.85 (0.82–0.88)	0.272	0.76	0.79	0.74	0.50	0.92	0.61
XGBoost	0.83 (0.80–0.86)	0.357	0.81	0.62	0.88	0.62	0.88	0.62

Abbreviations: AUC, area under the curve; DT, decision tree; Light GBM, light gradient boosting machine; LR, logistic regression; NPV, negative predictive value; PPV, positive predictive value; SVM, support vector machine; XGBoost, extreme gradient boosting.

### 3.4. Model Visualization and Interpretation

**FIGURE 3:**
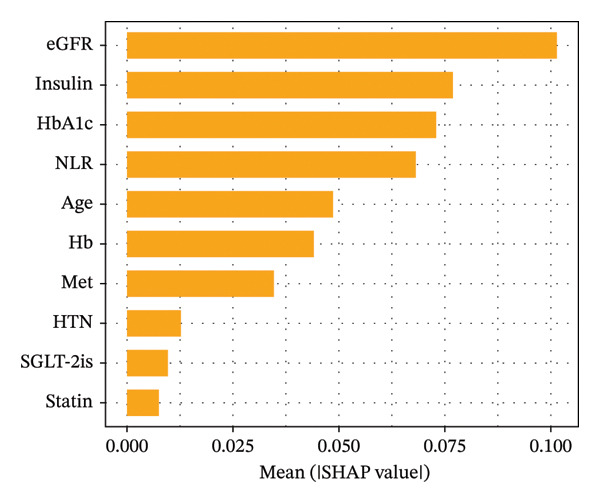
Feature importance matrix plot for Light GBM model. Abbreviations: SHAP, Shapley additive explanations; LightGBM, light gradient boosting machine; HbA1c, hemoglobin A1C; NLR, neutrophil‐to‐lymphocyte ratio; eGFR, estimated glomerular filtration rate; Hb, hemoglobin; HTN, hypertension; SGLT‐2is, sodium‐glucose cotransporter‐2 inhibitors; Met, metformin.

Following a comprehensive evaluation of model performance, this study ultimately selected the Light GBM model for further visual interpretation to explore feature importance within the predictive model. The SHAP method was employed to compute the contribution of each variable to the prediction outcomes. The importance matrix diagram indicates that the top 10 critical features for predicting MAU risk are, in order, eGFR, use of insulin, HbA1c, NLR, age, Hb, use of metformin, history of hypertension, use of SGLT‐2is, and use of statins (Figure 3).

**FIGURE 4:**
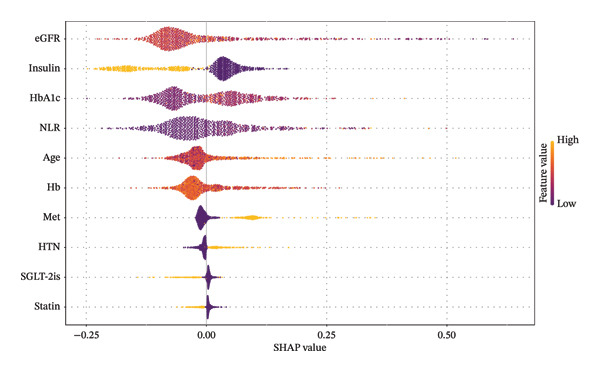
SHAP summary plot for LightGBM model. Abbreviations: SHAP, Shapley additive explanations; LightGBM, light gradient boosting machine; HbA1c, hemoglobin A1C; NLR, neutrophil‐to‐lymphocyte ratio; eGFR, estimated glomerular filtration rate; Hb, hemoglobin; HTN, hypertension; SGLT‐2is, sodium‐glucose cotransporter‐2 inhibitors; Met, metformin.

Figure 4 presents a SHAP summary plot, where the *y*‐axis ranks features based on the aggregate SHAP values across all samples, and the *x*‐axis displays the distribution of the impact of features on model output. The influence of features on prediction outcomes diminishes from top to bottom; each point represents a sample, with the color indicating the magnitude of the feature value—yellow for high values and purple for low values. Features with positive SHAP values indicate a positive impact on the prediction of T2DM progression to MAU, meaning these values lead to the occurrence of MAU in patients, with higher SHAP values denoting greater influence on the prediction outcomes. Generally, the features associated with an increased risk of MAU in T2DM patients include older age, poor glycemic control (high HbA1c), NLR > 3.7, Hb < 125 g/L, eGFR < 80 mL/min/1.73 m^2^, concurrent hypertension, and nonuse of insulin, SGLT‐2is, or statins.

The SHAP dependence plots in the model clearly display the impact of individual features on the prediction outcomes. A SHAP value greater than zero indicates an increased risk of MAU occurrence. Figures 5(a), 5(b), 5(c), 5(d), 5(e), 5(f), 5(g), 5(h), 5(i), and 5(j) sequentially represent the dependence plots for the top 10 critical features identified in this study for predicting MAU risk. The plots reveal that as the age feature value on the *x*‐axis increases, the distribution of points trends upwards, indicating that the risk of MAU occurrence increases with patient age. Specifically, when patient age exceeds 80 years, all data points are predominantly concentrated above the zero line of the SHAP values, signifying a notably higher risk of MAU under this condition.

FIGURE 5SHAP correlation graph of LightGBM model. (a) Age, (b) HbA1c, (c) NLR, (d) eGFR, (e) Hb, (f) HTN, (g) use of SGLT‐2is, (h) use of insulin, (i) use of metformin, and (j) use of statin. SHAP values above zero for specific characteristics indicate an increased risk of MAU. Abbreviations: SHAP, Shapley additive explanations; LightGBM, light gradient boosting machine; HbA1c, hemoglobin A1C; NLR, neutrophil‐to‐lymphocyte ratio; eGFR, estimated glomerular filtration rate; Hb, hemoglobin; HTN, hypertension; SGLT‐2is, sodium‐glucose cotransporter‐2 inhibitors; Met, metformin.

**Figure:**
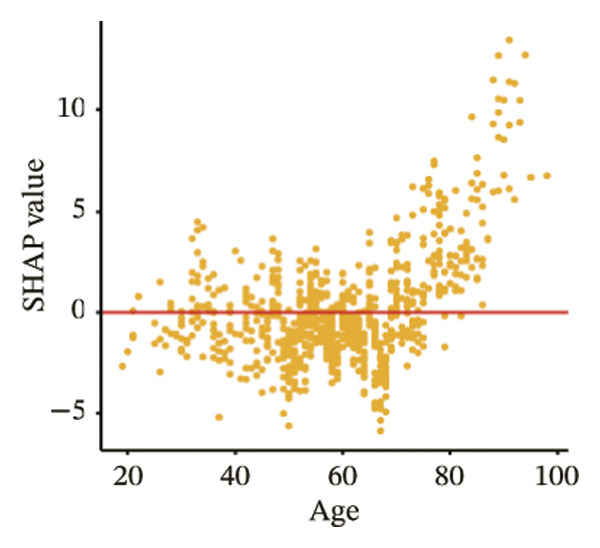
(a)

**Figure:**
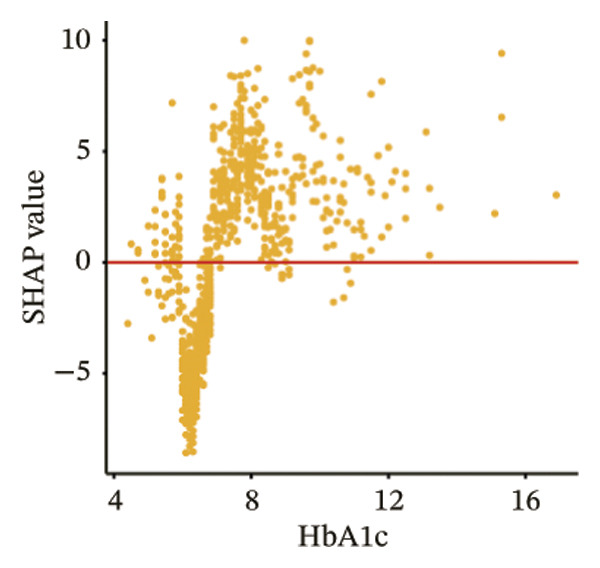
(b)

**Figure:**
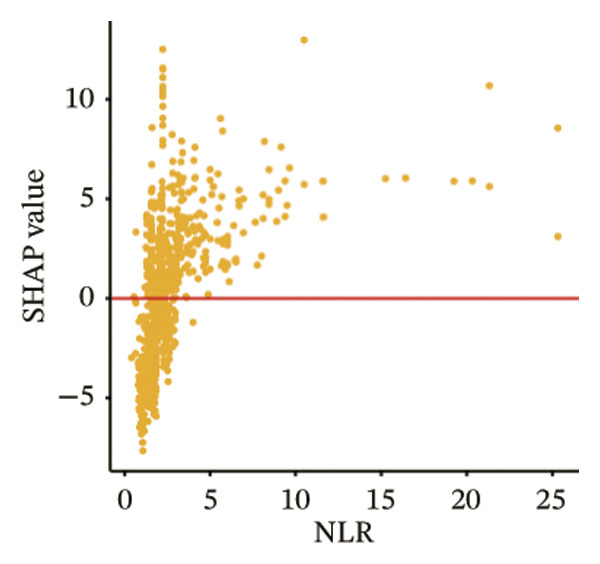
(c)

**Figure:**
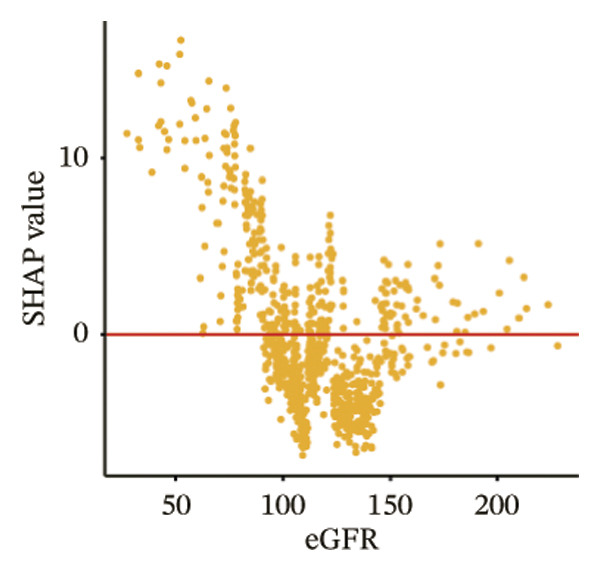
(d)

**Figure:**
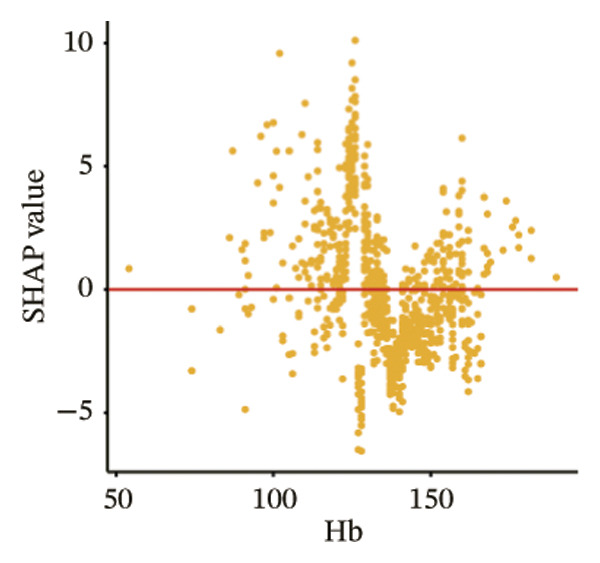
(e)

**Figure:**
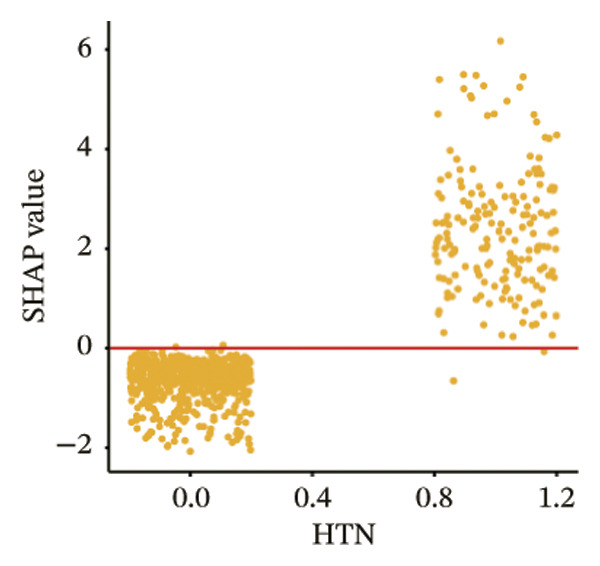
(f)

**Figure:**
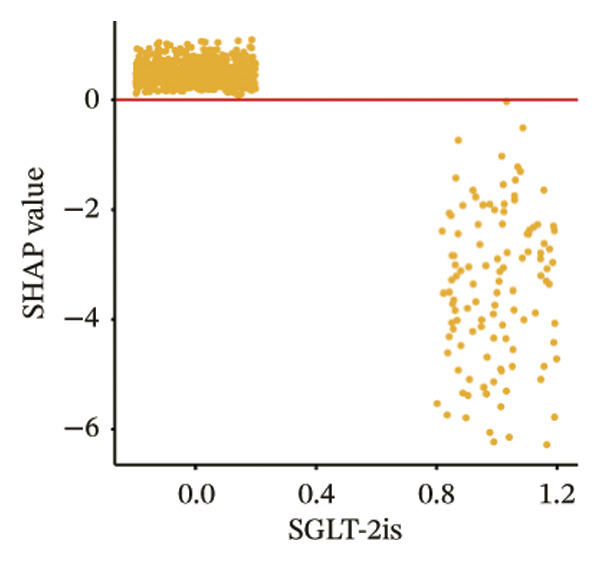
(g)

**Figure:**
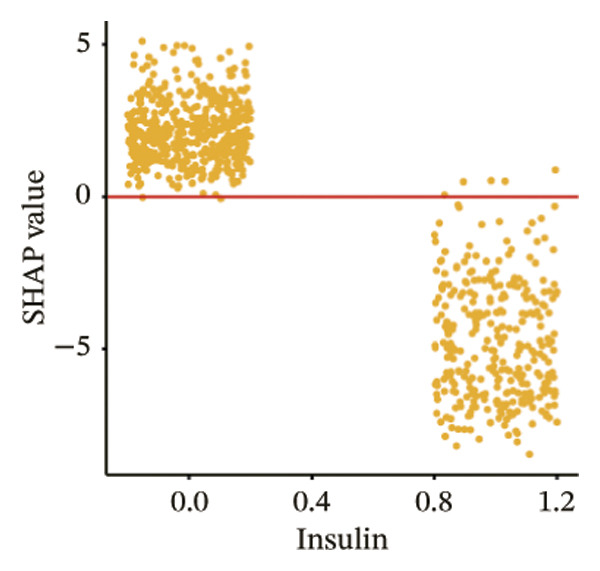
(h)

**Figure:**
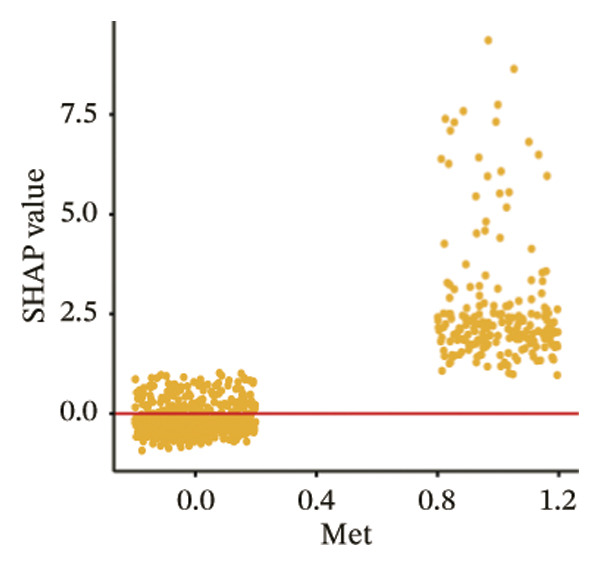
(i)

**Figure:**
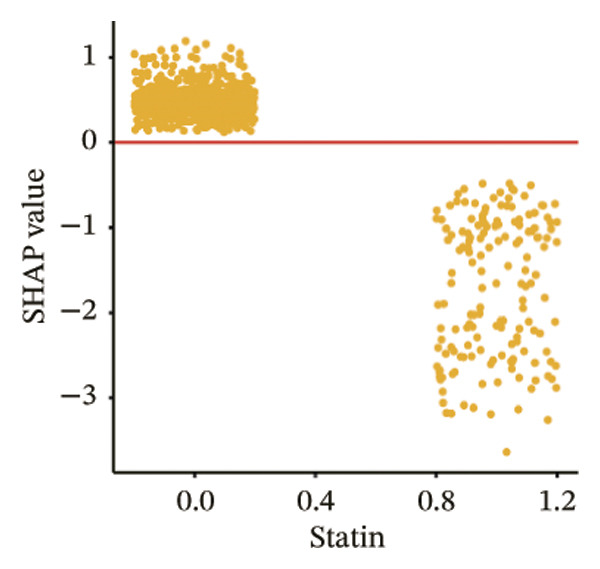
(j)

### 3.5. Individualized Risk Assessment

The SHAP force plots illustrate the impact of individual features on the predicted outcome for a single sample. Through the visual interpretation of the model described above, this study is able to categorize the population into three risk levels: high, medium, and low. Figures 6(a), 6(b), and 6(c) display individualized risk predictions for patients at high, medium, and low risk levels of MAU following diagnosis. These visualizations facilitate targeted interventions based on the stratified risk profiles, offering a refined approach to managing and predicting MAU risks effectively. Figure 6(a) illustrates the characteristics contributing to a high risk of MAU (probability of 90.6%): an older age of 78, history of hypertension, HbA1c at 8.1%, eGFR at 69.9 mL/min/1.73 m^2^, NLR at 21.3, and nonuse of insulin, SGLT‐2is, and statins. Each of these features has a varying degree of positive impact on the outcome, with eGFR having the most significant positive influence, whereas the nonuse of metformin has a negative impact. For the patient depicted in Figure 6(b) with a medium risk of MAU (probability of 51.8%), high HbA1c levels (8.1%), older age, and high NLR values are the main drivers increasing the risk of MAU, with a normal eGFR value acting as a negative influencer. For the patient in Figure 6(c) at low risk of MAU (probability of 26.2%), the model identifies normal eGFR values, better HbA1c levels, and younger age as the primary reasons for the low risk of MAU.

FIGURE 6SHAP force plot for patients in the dataset at different risk of developing MAU. (a) Patients at high risk, (b) patients at median risk, and (c) patients at low risk. Abbreviations: SHAP, Shapley additive explanations; HbA1c, hemoglobin A1C; NLR, neutrophil‐to‐lymphocyte ratio; eGFR, estimated glomerular filtration rate; Hb, hemoglobin; HTN, hypertension; SGLT‐2is, sodium‐glucose cotransporter‐2 inhibitors; Met, metformin.

**Figure:**
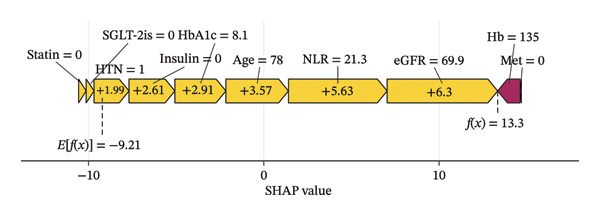
(a)

**Figure:**
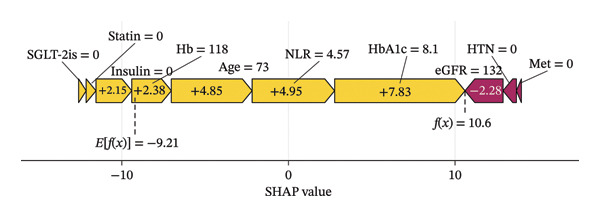
(b)

**Figure:**
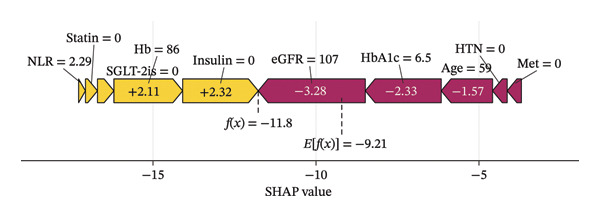
(c)

## 4. Discussion

This study developed and compared five ML algorithms for constructing predictive models to assess the risk of MAU in T2DM patients. Patients’ age at the baseline, Hb, NLR, HbA1c, eGFR, history of hypertension, and the utilization of antidiabetic and statin medications were identified as feature variables in the predictive models. SHAP plots were utilized to visually interpret the best‐performing Light GBM model, further elucidating the contribution of each feature to the overall population risk. This methodological approach enhances our understanding of the factors influencing MAU development, allowing for more targeted risk management and therapeutic strategies for T2DM patients.

Traditional statistical approaches like LR or Cox regression are often limited by linear postulates, the assumption of feature independence, and an insufficient capacity to handle complex data, making it difficult to capture multidimensional nonlinear relationships and interactive effects. ML overcomes these limitations by providing higher prediction accuracy and automated feature selection capabilities. Light GBM, a high‐performance gradient boosting framework‐based ML model, has significant advantages in ML tasks with large data volumes and high‐dimensional features, and it has been used in studies predicting undiagnosed T2DM based on electronic medical records (EMR) [17–19].

In our model, effective control of blood glucose, blood pressure, and lipids has a positive impact on preventing the onset and progression of T2DM. Numerous studies confirm [20–22] that poor glucose control is a key factor in promoting the occurrence of proteinuria. According to the Action in Diabetes and Vascular Disease: Preterax and Diamicron MR Controlled Evaluation (ADVANCE) report, intensive glycemic control that lowers HbA1c below 6.5% can reduce the incidence of new MAU by 9% and decrease the relative risk of nephropathy by 21% [23]. This study also found that patients with a baseline HbA1c exceeding 7% are at a significantly heightened risk of MAU, especially when HbA1c approaches 8%, marking a peak in the risk of MAU.

SHAP analysis of our model reveals that features closely related to renal function, such as the presence of hypertension and eGFR values, are significant predictors of MAU. A decline in eGFR indicates reduced glomerular filtration capability, which directly results in a higher propensity for proteins to leak into the urine. Hypertension can cause changes in renal intravascular hemodynamics, leading to glomerular hypertension and hyperfiltration [24], which indirectly increases protein excretion in the urine. Studies have shown that effective blood pressure control and the use of RAAS inhibitors can reduce the incidence of proteinuria and the progression of end‐stage renal diseases [25, 26]. Additionally, a prospective clinical cohort study involving 15,517 subjects over a 26‐year follow‐up period demonstrated that diabetes is a significant risk factor for renal function decline, with the rate of eGFR decrease in diabetic patients being nearly twice that of nondiabetics [27]. Moreover, when hypertension and diabetes coexist, there is a marked increase in the risk of cardiovascular and renal terminal organ damage, leading to higher rates of cardiovascular events and overall cardiovascular mortality [28]. As patients age, their renal function gradually deteriorates, and older patients often have a longer duration of diabetes, which may explain the trend observed in the SHAP dependence plot for age, indicating that the risk of MAU in T2DM patients increases with age.

In recent years, novel antidiabetic drugs, specifically SGLT‐2is, have demonstrated pronounced advantages in improving cardiac and renal functions in patients with T2DM. Notably, the CREDENCE trial [29] was the first to report a definitive benefit of SGLT‐2is on renal outcomes. The results of the CANVAS trial [30] indicated that SGLT‐2is significantly slow the progression of proteinuria, consistent with the findings of this study that baseline use of SGLT‐2i is a protective factor against the occurrence of MAU in patients with T2DM. The study did not find a significant impact of baseline use of metformin on the risk of MAU occurrence, whereas baseline use of insulin was associated with a marked reduction in the risk of MAU, possibly due to better glycemic control in patients treated with insulin.

Previous research has shown that elevated levels of TG and LDL‐C [31, 32] promote the occurrence of dyslipidemia. Dyslipidemia may lead to structural changes in the glycosaminoglycan (GAG) of the renal basement membrane [33], and abnormalities in GAG structure are one of the direct causes of weakened basement membrane barrier function, leading to protein leakage. Additionally, LDL‐C can bind to LDL receptors on the membranes of renal mesangial cells, inflicting damage upon both mesangial and podocyte cells, exacerbating proteinuria, and advancing the progression of glomerulosclerosis and interstitial fibrosis [34]. This study found that the incidence of MAU was significantly reduced in patients who were administered statins at baseline, suggesting that statins may protect against the onset of MAU by lowering LDL‐C levels, which in turn mitigates renal lipid deposition and associated glomerular damage.

Our visualization results from the model further elucidated significant contributions of the NLR and Hb to the occurrence of MAU in patients with T2DM. NLR is considered a reliable indicator of systemic inflammation and is associated with inflammation induced by metabolic syndrome and insulin resistance [35]. The conclusion that NLR levels are independently associated with a decline in eGFR and an increase in urinary protein excretion has been confirmed in previous studies [35, 36]. Serum Hb levels have been shown, in real‐world studies, to be inversely correlated with all renal pathological characteristics, particularly the severity of interstitial fibrosis, even after adjusting for known risk factors for the progression of diabetic kidney disease (correlation coefficient = −0.52; *p* < 0.001) [37]. Additionally, low Hb levels (anemia) are more common among diabetic patients than in nondiabetics [38].

Previous studies specifically focused on the prediction of MAU in patients with T2DM remain limited. Wei et al. [39] developed a regression‐based prediction model for MUA using data from 3294 Chinese individuals, achieving moderate predictive performance. More recently, Long et al. [40] applied two ML approaches in a cohort of 981 patients with T2DM and reported favorable discrimination after addressing class imbalance using synthetic oversampling techniques. In comparison, while the discrimination performance of our model is comparable, it was developed in a significantly larger real‐world cohort and explicitly incorporated glucose‐lowering treatment strategies, which may enhance robustness and improve the applicability of risk stratification in contemporary diabetes management.

This study still has several limitations worthy of discussion. First, although based on a large sample size and employing a robust internal validation approach, its single‐center design may restrict the generalizability of the model across populations with varying geographic regions and healthcare practice patterns [41, 42]. These variations may partly explain the heterogeneity in model performance across populations and highlight the need for future validation across multicenter datasets and diverse clinical contexts. Second, participants were recruited from a tertiary referral center, where patients typically present with more complex disease conditions and relatively standardized management protocols. This may introduce selection bias, necessitating caution when extrapolating the findings to primary care or community‐based populations. Additionally, constrained by the availability of retrospective data, certain potential confounding factors were not captured in this analysis, such as dietary habits [43, 44] and molecular markers related to renal susceptibility [45], which should be investigated in future prospective studies to further elucidate their influence.

## 5. Conclusion

Our study constructed an efficient and robust ML model, and the selected Light GBM model achieved an AUC of 0.85. This model effectively utilizes baseline data to predict the risk of MAU in patients with T2DM. Feature importance ranking analysis indicated that key risk drivers or related characteristics for the occurrence of MAU include baseline age, Hb levels, NLR, HbA1c, eGFR, presence of hypertension, use of antidiabetic drugs, and statins. Through SHAP analysis, we explained the extent of impact of each factor on the predicted outcomes at both the cohort and individual levels. Additionally, the population was stratified into distinct risk categories based on risk probability values, aiming to tailor personalized intervention and treatment plans for patients at varying risk strata.

## Author Contributions

Haixia Zhang and Cheng Ji: conceptualization, funding acquisition, and project administration. Zixuan Liu and Zhuolin Zhou: data curation, formal analysis, methodology, visualization, writing–original draft, and writing–review and editing. Yu Sun and Xiaotian Du: investigation and validation.

## Funding

This work was supported by the Funding for Clinical Trials from the Affiliated Drum Tower Hospital, Medical School of Nanjing University (grant number 2021‐LCYJ‐PY‐33), and the Project of Modern Hospital Management and Development Institute, Nanjing University (grant number NDYG2022049).

## Disclosure

All authors have seen and approved the final version of the manuscript being submitted. The article is the authors’ original work, has not received prior publication, and is not under consideration for publication elsewhere.

## Ethics Statement

The protocol for this research project has been approved by the Medical Ethics Committee of Nanjing Drum Tower Hospital (grant numbers: 2021‐403‐02). Throughout the entirety of the study, ethical principles and the Helsinki Declaration were strictly adhered to, with their personal privacy and rights protected.

## Consent

Informed consent was obtained from all subjects and/or their legal guardian for anonymized patient information to be published in this article.

## Conflicts of Interest

The authors declare no conflicts of interest.

## Data Availability

The data that support the findings of this study are available from specialized diabetes database of the Nanjing Drum Tower Hospital, but restrictions apply to the availability of these data, which were used under license for the current study, and so are not publicly available. Data are however available from the corresponding author upon reasonable request and with permission of the Nanjing Drum Tower Hospital.
